# MicroRNA-122 in human cancers: from mechanistic to clinical perspectives

**DOI:** 10.1186/s12935-023-02868-z

**Published:** 2023-02-20

**Authors:** Mahboobeh Faramin Lashkarian, Nasrin Hashemipour, Negin Niaraki, Shahrad Soghala, Ali Moradi, Sareh Sarhangi, Mahsa Hatami, Fatemehsadat Aghaei-Zarch, Mina Khosravifar, Alireza Mohammadzadeh, Sajad Najafi, Jamal Majidpoor, Poopak Farnia, Seyed Mohsen Aghaei-Zarch

**Affiliations:** 1 Department of Molecular Genetics, Central Tehran Branch, Islamic Azad University, Tehran, Iran; 2 Department of Biology, School of Science, Yazd University, Yazd, Iran; 3 Department of Cell and Molecular Biology, School of Life Sciences and Biotechnology, Shahid Beheshti University, Tehran, Iran; 4 Department of Biology, Science and Research Branch, Islamic Azad University, Tehran, Iran; 5 School of Advanced Sciences and Technology, Tehran Medical Sciences Branch, Islamic Azad University, Tehran, Iran; 6 Department of Genetics, School of Advanced Sciences and Technology, Tehran Medical Sciences Branch, Islamic Azad University, Tehran, Iran; 7 Department of Medical Genetics, School of Medicine, Shahid Beheshti University of Medical Sciences, Tehran, Iran; 8 School of Medicine, Kerman University of Medical Sciences, Kerman, Iran; 9 Institute of Diabetes, Obesity, and Metabolism, Perelman School of Medicine, University of Pennsylvania, Philadelphia, PA 19104 USA; 10 Department of Microbiology, School of Medicine, Infectious Diseases Research Center, Gonabad University of Medical Sciences, Gonabad, Iran; 11 Department of Medical Biotechnology, School of Advanced Technologies in Medicine, Shahid Beheshti University of Medical Sciences, Tehran, Iran; 12 Department of Anatomy, Faculty of Medicine, Infectious Disease Research Center, Gonabad University of Medical Sciences, Gonabad, Iran; 13 Mycobacteriology Research Centre, National Research Institute of Tuberculosis and Lung Disease, Shahid Beheshti University of Medical Sciences, Tehran, Iran

**Keywords:** Cancer, miR-122, Pathogenesis, Therapy, Prognosis

## Abstract

MicroRNAs (miRNAs) are endogenous short non-coding RNAs that can regulate the expression of target genes post-transcriptionally and interact with mRNA-coding genes. MiRNAs play vital roles in many biological functions, and abnormal miRNA expression has been linked to various illnesses, including cancer. Among the miRNAs, miR-122, miR-206, miR-21, miR-210, miR-223, and miR-424 have been extensively studied in various cancers. Although research in miRNAs has grown considerably over the last decade, much is yet to be discovered, especially regarding their role in cancer therapies. Several kinds of cancer have been linked to dysregulation and abnormal expression of miR-122, indicating that miR-122 may serve as a diagnostic and/or prognostic biomarker for human cancer. Consequently, in this review literature, miR-122 has been analyzed in numerous cancer types to sort out the function of cancer cells miR-122 and enhance patient response to standard therapy.

## Introduction

MicroRNAs (miRNAs) are small noncoding RNAs of approximately 22 nucleotide (nt) that are known to have a significant role in the regulation of the post-transcriptional level of messenger RNAs (mRNAs) [1, 2]. MiRNAs are 22 nt noncoding RNAs recognized to play a crucial function in the post-transcriptional control of mRNA [3–5]. MiRNAs are usually generated from transcripts of incipient primary miRNAs (pri-miRNAs) through two successive hairpin-shaped precursors (pre-miRNAs) [6]. Pre-miRNAs are transported from the nucleus to the cytoplasm by exportin 5 (XPO5) and cleaved by DICER. The ensuing small RNA duplexes are loaded with Argonaute (AGO) proteins that favorably conserve only one strand of the mature miRNA while taking out the other strand. Together with other molecules like GW182, AGO that has been loaded with miRNA may produce an effector complex known as the RNA-induced silencing complex (RISC). RISC triggers translational repression by interacting with complementary sequences in the 3ʹ-untranslated region (3ʹ-UTR) of target gene mRNAs [7]. As a consequence of this, variations in the levels of miRNA expression have a significant influence on human health as well as the progression of diseases such as cancer. In this regard, Volinia et al. conducted a microarray study and found that miRNAs play a significant role in the carcinogenic process of solid tumors. These findings provide evidence for the function of miRNAs as either dominant or recessive cancer genes [8].

Initial analysis of miR-122 revealed it to be conserved in 12 different species, including humans, frogs, and zebrafish [9]. Since September 2011, the miR-122 expression has been investigated in 18 species, which are all vertebrates. In humans, miR-122 originates from a single genomic region located on chromosome 18. The human miR-122 locus is located in an isolated region of noncoding RNA exons. The pri-miR-122 gene was discovered initially as hcr, a noncoding RNA, and was recently described in depth. The 7.5 kb precursor transcript is created during pri-miR-122 synthesis, and it is subsequently spliced to form the 4.5 kb pri-miR-122, as shown by the localization of the transcription start site and 3' end. The miR-122 core promoter is very well-conserved and has characteristics indicative of a pol II promoter. The liver-enriched transcription factor hepatocyte nuclear factor 4 (HNF-4), which promotes miR-122 production, has a conserved target location there. In the adult liver, miR-122 is expressed at around 66,000 copies per cell, making it one of the most abundant miRNAs in the body [10]. There are no known paralogs of the mature form of miR-122, and its sequence is conserved across all species where it has been found, suggesting that its entirety is crucial to its function. The very liver-specific expression pattern of miR-122 was also shown to be retained across multiple species when it was investigated using in situ hybridization in zebrafish [11].

MiR-122 is involved in a broad range of cellular processes and interacts with many other molecules [12]. Hepatocyte nuclear factor 4A (HNF4A) is a transcription factor that has been shown to control miR-122 via targeting NF-кB-inducing kinase (NIK). Evidence is shown that HCV infection causes an upregulation of NIK expression and downregulation of HNF4A and miR-122 [13]. It has been established by Long et al. that miR-122 inhibition protects hepatocytes against lipid metabolic disorders like Non-alcoholic fatty liver disease (NAFLD) by decreasing lipogenesis and enhancing Sirt1 and AMP-activated protein kinase (AMPK) activity [14]. Additionally, ZW Zhang et al. investigated miR-122’s function in cardiomyocyte apoptosis and found that it has an inductive influence in this process, perhaps because of its influence on caspase-8 [15].

**Fig. 1:**
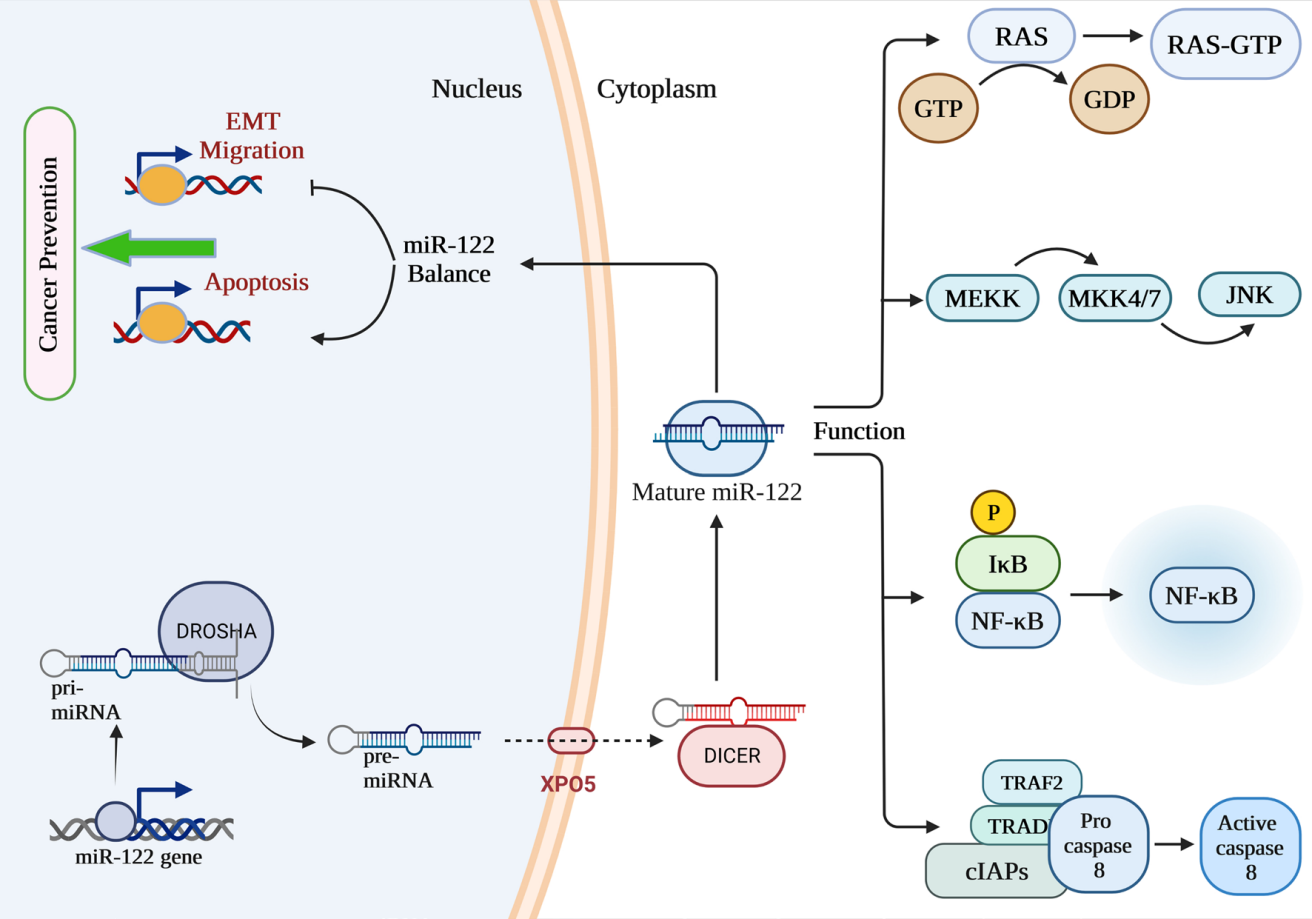
Schematic representation of miR-122 biosynthesis and its role in cancer progression

MiRNAs have emerged as a new cell component with variable expression in human cancers throughout the last several years of study. Among the miRNAs, miR-122, miR-206, miR-21, miR-210, miR-223, miR-34a, miR-22, miR-25, miR-320, miR-150, miR-200c, miR-451 and miR-424 have been extensively studied in various cancers [16, 17]. Targeting a variety of genes in human malignancies, miR-122 has the potential to act either as an oncogene or a tumor suppressor. MiR-122 is aberrantly expressed in various tumors, including breast cancer, lung cancer, bladder, leukemia, liver cancer, colorectal cancer, ovarian cancer, and esophageal, acting as both a tumor promoter and tumor silencer. Furthermore, miR-122’s deregulation and aberrant expression in carcinogenesis and tumor development of various cancer types suggest it may be helpful as a diagnostic and/or prognostic marker for human cancer. The miR-122 may also increase the sensitivity of tumor cells to chemotherapy agents. In light of these results, miR-122 is a potential novel therapeutic target and diagnostic/prognostic molecular marker for cancer. As a result, the present review offers a thorough introduction to miR-122 and its role in human cancers, shedding light on this role and assisting in the development of targeted therapeutics (Fig. 1).

## Mechanism of miR-122 in human cancers

### *Digestive* system

#### Liver cancer

The second most significant cause of cancer-related fatalities globally is hepatocellular carcinoma (HCC). Therefore, a more in-depth knowledge of the molecular abnormalities pertinent to HCC etiology is essential for creating a viable treatment. By examining RNA-seq data and clinical information from The Cancer Genome Atlas, JM Barajas et al. evaluate the clinical importance of Glucose-6-phosphate dehydrogenase (G6PD) and miR-122 in primary human HCC. They show that the expression of miR-122 is inversely correlated with G6PD expression and that G6PD mRNA levels increase in tandem with rising tumor grade in The Cancer Genome Atlas liver cancer patients. Additionally, they found three miR-122 binding sites in G6PD’s 3′UTR region and used a luciferase reporter test to verify two of the conserved sites. Their discovery demonstrates that miR122’s anti-HCC activity may be at least partially mediated by inhibiting PPP via reducing the expression of G6PD [18]. In addition, Li et al. uncovered the function of miR-122 in the development of hepatocarcinoma. First, they discovered that miR-122 levels were lower in hepatocarcinoma tissues than in paracarcinoma tissues. Additionally, they discovered that overexpression of miR-122 might prevent cell proliferation, migration, or invasion in vitro, which was in line with earlier research. Lamin B2 (LMNB2), a biomarker for carcinogenesis in hepatocarcinoma, was shown to be a direct target of miR-122 by their findings. They concluded that miR-122-LMNB2 might be a therapeutic target for hepatocarcinoma and that abnormal changes between miR-122 and LMNB2 can be connected with the advancement of hepatocarcinoma [19]. It was demonstrated that Apolipoprotein B mRNA editing enzyme catalytic polypeptide 2 (APOBEC2) selectively alters the Eif4g2 and perhaps the PTEN genes' nucleotide sequences, and its constitutive expression in epithelial tissues promotes the growth of several malignancies, including HCC [20]. Li et al. looked into the molecular connections between liver cancer development, miR-122, APOBEC2 expression, and HBV infection. It was discovered that HBV significantly increased the amount of APOBEC2 expression in hepatocytes and that miR-122 inhibits this expression by binding to the 3′UTR of APOBEC2 mRNA. Additionally, it was proposed that HBV accelerated the growth of liver cancer by promoting the expression of APOBEC2 by downregulating cellular miR-122 [21]. Furthermore, HOTAIR and miR-122 were studied in HCC by Cheng et al. They discovered that while miR-122 was repressed in HCC, HOTAIR was substantially elevated. Mechanistically, the miR-122 promoter region included a CpG island, and HOTAIR epigenetically reduced miR-122 production via DNA Methyltransferases (DNMTs)-mediated DNA methylation. They concluded that Cyclin G1 activation and increased tumorigenicity in HCC are caused by DNA methylation-mediated suppression of miR-122 [22]. Moreover, it was discovered by HM Lee et al. that elevated blood levels of miR-122-5p were individually associated with a higher risk of recurrent liver cancer (LC) in individuals with type 2 diabetes (T2D) and may be valuable indicators for the early diagnosis of LC in people with type 2 diabetes [23].

#### Gastric cancer

One of the malignant tumors with the highest morbidity and mortality rates worldwide is gastric cancer (GC). Despite significant advances in surgical and chemotherapy techniques over the past few decades, the 5-year overall survival (OS) rate of GC patients remains poor. Finding promising targets for the treatment and detection of different phases of GC is thus crucial. In this context, Meng et al. looked at the biological role of miR-122-5p in GC. They discovered that miR-122-5p had low expression levels in GC tissues and cells and prevented GC cells from proliferating, migrating, and invading by suppressing the expression of LYN [24]. MiR-122-5p and Dual Specificity Phosphatase 4 (DUSP4) were investigated concerning their impact on gastric cancer (GC) cell motility and invasiveness by Xu et al. They established the miR-122-5p binding sites on the 3′UTR of DUSP4 and the target connection between miR-122-5p and DUSP4 using bioinformatics prediction tools and dual luciferase reporter experiment. They concluded that miR-122-5p reduced DUSP4 levels, which in turn reduced GC cell motility, invasiveness, and pulmonary tumor metastasis [25]. In addition, Rao et al. found that the expression of miR-122 was significantly reduced in GC tissues and cell lines. This reduction was associated with aggressive clinicopathological features in patients. Further investigation revealed that GC cell line proliferation, migration, and invasion were severely suppressed by miR-122 overexpression caused by direct targeting of cAMP response element binding protein 1 (CREB1) [26]. Additionally, Pei et al. have recently looked at the relationship between miR-122-5p expression and cell growth and apoptosis in a GC cell line. They used experimental research to demonstrate that miR-122-5p caused cell growth arrest in SCG7901 cells by upregulating p27 expression. However, studies on cell deaths revealed that miR-122-5p caused apoptosis in SCG7901 cells by targeting MYC [27]. S Maruyama et al. also investigated the molecular mechanism behind tumor growth in Fetoprotein (AFP)-producing gastric cancer (AFPGC), as well as the biological role of miR-122-5p in this disease. Additionally, Maruyama et al. investigated the natural role of miR-122-5p and the molecular mechanisms behind tumor growth in gastric cancer that produces-Fetoprotein (AFP) (AFPGC). In AFPGC, miR-122-5p suppressed apoptosis and accelerated tumor growth via targeting Forkhead box O3 (FOXO3), suggesting that miR-122-5p may be a potential therapeutic target in AFPGC [28].

#### Bile duct carcinoma

With a median survival time of fewer than 2 years, bile duct cancer (BDC), which is sometimes referred to as cholangiocarcinoma (CCA), is the second most frequent kind of primary hepatic carcinoma. Over time, survival and treatment options for cholangiocarcinoma have proven poor and have not improved. Because of this, there is an immediate need to investigate the pathophysiology of this malignancy in order to get a better understanding of BDC and explore helpful therapies. Xu et al. aimed to learn more about the roles and processes of Aldolase A (ALDOA) in bile duct cancer and whether or not miR-122-5p might be used to modulate cell proliferation and death. Using bioinformatics and clinical samples, they demonstrated the inverse relationship and targeted modulation between miR-122-5p and ALDOA. It was discovered that downregulating ALDOA caused overexpression of miR-122-5p to induce cell apoptosis, drastically decrease cell proliferation and invasion, and suppress the formation of tumors in vivo [29]. Additionally, Kong et al. found that lncRNA-UCA1 encouraged the metastasis of BDC cells via controlling miR-122/CLIC1 and activating the Mitogen-activated protein kinase/extracellular signal-regulated kinase (ERK/MAPK) signaling pathway, according to bioinformatics analysis and in vitro tests [30]. Zhu et al. also aimed to learn more about the function of HNF6, particularly the molecular processes by which HNF6 contributes to the development and metastasis of CCA cells. These findings suggest overexpressing HNF6 may be a mechanism-based treatment for CCA since it modulates miR-122 to function as a tumor suppressor [31].

#### Colon/Colorectal cancer

With 1.36 million new cases per year, colorectal cancer (CRC) is the third most prevalent cancer in the world. Additionally, W Yin et al. [32] investigated whether circ_0007142 might control CRC development through the miR-122-5p/ Cell Division Cycle 25A (CDC25A) axis. They discovered that Circ_0007142 accelerated the development of CRC by controlling CDC25A expression through miR-122-5p. The advancement of CRC caused by the circ 0007142/miR-122-5p/CDC25A gene shed information on the disease’s pathogenesis and offered new diagnostic indicators for CRC patients. Patients with CRC typically die from metastasis. Liquid biopsies, which may be taken continuously, may one day help in predicting recurrence and metastatic development. Sun et al. sought new circulating exosomal miRNAs that might indicate liver-metastasized (LM) colorectal cancer. The combined study of three GEO datasets and clinical samples revealed that tissues from CRC patients had miR-122 overexpression. Then, their exosome sequencing confirmed that the higher serum miR-122 originated from tumors. CRC patients, especially those with LM, were also shown to have elevated blood exosomal miR-122 expression. Thereby, Serum exosomal miR-122 could consequently represent a novel potential predictive and diagnostics biomarker in CRC patients with LM [33]. Importantly, using bioinformatics methods, Li et al. reexamined the available data to locate differentially expressed microRNAs in colon cancer cells. Their findings demonstrated that miR-122 greatly enhances SW480 and SW620 cells’ capacity for growth and penetration by suppressing ALDOA expression [34].

#### Esophageal cancer

Due to the high incidence and poor prognosis of esophageal cancer, research into potential pathways is urgently needed. Recent research suggests that the Kinesin Family Member 22 (KIF22)/miR-122 axis might serve as a biomarker for esophageal squamous cell carcinoma (ESCC) by reducing miR-122 levels, inhibiting the KIF22-mediated growth of ESCC cells [35]. By inhibiting PKM2 expression through up-regulation of miR-122, Zhang et al. sought to explore the anticancer effect of tanshione IIA in human esophageal cancer Ec109 cells. Their results prove that tanshione IIA inhibits Ec109 cell growth by controlling pyruvate kinase isozymes M2 (PKM2) expression. The metabolic switch in Ec109 cells was controlled by miR-122, which is upregulated when PKM2 expression is downregulated. This results in a compensatory downregulation of the central and final enzyme in glycolysis, inhibiting Ec109 cell growth [36]. In conclusion, miR-122 is vital for human esophageal cancer cells.

#### Gallbladder cancer

One of the deadliest illnesses of the digestive system is gallbladder cancer (GBC). Due to the large percentage of advanced tumors at the time of presentation, the prognosis for GC is typically very poor. The median survival for people with presumed tumors is 9.2 months, while for those with cancers unexpectedly discovered, it is 26.5 months. The 5-year prognosis for all stages of GCs is approximately 5%. Therefore, novel signaling pathways in GBC should be investigated to develop new molecular prognostic markers and therapeutic targets for gallbladder cancer. Lu et al. looked into the possible function and further use of miR-122 in GBC to assess the expression control and anticancer properties in gallbladder cancer. Exogenous miR-122 has been shown to suppress GBC cell line multiplication, motility, and invasion by inhibiting epithelial-mesenchymal transition (EMT) by affecting PKM2. This indicates that increasing the expression of miR-122 in GBC could be a potential treatment for GBC [37].

#### Nasopharyngeal carcinoma

Nasopharyngeal carcinoma (NPC) has one of the highest prevalence rates of all cancers in endemic areas. Head and neck cancer, or NPC, has a poor prognosis and is uncommon in most of the world but more prevalent in particular locations, such as southern Asia and parts of North East India (Nagaland, Manipur, and Mizoram). The mechanism underlying NPC progression has not been thoroughly investigated or defined. Therefore, further research is advantageous to enhance survival in patients whose NPC is in the early stages, particularly for identifying relevant indicators. The goal of Liu et al. was to investigate the miR-122-5p expression and functional action in NPC. According to their findings, miR-122-5p expression levels were considerably reduced in NPC cell lines. Furthermore, it was shown that miR-122-5p targeted the particular AT-rich sequence-binding protein 1 (SATB1). Moreover, they found that miR-122-5p inhibits a wide range of cell functions by targeting SATB1, including colony formation, cell invasion, cell proliferation, and cell migration. This suggests that miR-122-5p may be helpful as a therapeutic target for NPC due to its tumor-suppressive function [38].

### Reproductive system

#### Breast cancer

Breast cancer (BC) is a significant threat to public health since it is one of the most aggressive forms of cancer. Elucidating the mechanisms underlying breast tumorigenesis and progression is beneficial for clinical management. Finding new, efficient therapeutic targets for breast cancer is thus urgently needed for women with breast cancer. The role of extracellular miR-122 in the development and metastasis of breast cancer was studied by Fong et al. They show that by secreting vesicles with elevated amounts of miR-122, cancer cells may stop non-tumor cells in the pre-metastatic niche from taking glucose. Breast cancer patients who have metastasized had high levels of miR-122 in their blood, which suggests that miR-122 is produced by cancer cells and promotes metastasis by making more nutrients available in the premetastatic niche. In vitro and in vivo, miR-122 from cancer cells inhibits niche cells’ glucose intake via reducing pyruvate kinase, a glycolytic enzyme. In addition, inhibiting miR-122 in vivo restores glucose absorption in distant organs, including the brain and lungs, which reduces the risk of metastasis. These results show that extracellular miR-122 released from cancer cells may change systemic energy metabolism to promote disease progression by altering glucose use by recipient premetastatic niche cells [39]. Wang et al. have done significant original work to understand the molecular process underlying triple-negative breast cancer (TNBC) progression better and identify potential TNBC gene therapy targets. They looked at the effects of miR-122-binding 5p’s on CHMP3’s 3′-UTR in TNBC cells using various in vitro analyses. Their study found that when comparing TNBC cells to normal cell lines, miR-122-5p was considerably elevated, and the CHMP3 gene dramatically downregulated. Additionally, upregulating the CHMP3 gene had the opposite effect from miR-122-5p mimics, which increased TNBC cell survival, proliferation, and invasion. They concluded that miR-122-5p, via inhibiting CHMP3 through MAPK signaling, increases aggressiveness and EMT in TNBC [40]. By modulating the Phosphoinositide 3-kinase/Akt/mammalian target of rapamycin/p70S6 kinase (PI3K/Akt/mTOR/p70S6K) pathway and targeting insulin like growth factor1 receptor (IGF1R), Wang’s et al. research showed that miR-122 acts as a tumor suppressor and is crucial in preventing the growth of new tumors. These factors suggest that miR-122 could be a novel therapeutic or diagnostic/prognostic target for the treatment of BC [41]. Additionally, CDKN2B-function AS1’s and mechanisms in human breast cancer were studied by Qin et al. They demonstrated that CDKN2B-AS1 regulated serine/threonine kinase 39 (STK39) expression by acting as a miR-122-5p sponge, aiding breast cancer advancement. They also discovered that miR-122-5p modified STK39 expression to regulate the impact of sh-CDKN2B-AS1 [42].

#### Ovarian cancer

One of the most prevalent gynecologic malignancies, ovarian cancer, has a high mortality and morbidity rate. Most OC patients were discovered at a late and incurable stage because the disease developed asymptomatically, and no early diagnosis or screening tools were available. To improve the early detection and treatment of OC patients, it is necessary to look for innovative biomarkers. MiR-122 activity in ovarian cancer cells was examined by Duan et al. who also looked at the underlying process. For the first time, they revealed that miR-122 suppressed ovarian cancer cell motility, invasion, EMT, and metastasis in the peritoneal cavity by targeting prolyl 4-Hydroxylase Subunit Alpha 1 (P4HA1), shedding light on the identification of miR-122 and P4HA1 as prospective diagnostic indicators and therapeutic targets for OC [43]. Additionally, X Huang et al. investigated circ 0072995, miR-122-5p, and SLC1A5 expression patterns in OC tissues and their impacts on cell proliferation, migration, invasion, and apoptosis. By knocking down miR-122-5p, the effects of circ_00729995 silence on OC cell progression were reversed, and it was found miR-122-5p to be a direct target of circ_0072995. Further investigation discovered that miR-122-downstream 5p’s target gene was SLC1A5 and that miR-122-5p overexpression prevented the development of OC cells by specifically targeting SLC1A5. As a result, the axis of circ 0,072,995/miR-122-5p/SLC1A5 is implicated in the malignant progression of OC and suggests a possible target for OC treatment [44].

#### Bladder cancer

A common disease from the bladder’s epithelial lining is bladder cancer. Critical molecular pathways of miR-122 in bladder carcinogenesis and angiogenesis were studied by Wang et al. They found a previously unknown component of bladder cancer development, a connection between miR-122 and Vascular Endothelial Growth Factor C (VEGFC). MiR-122 overexpression slowed the development of bladder cancer cells as a tumor suppressor. Their findings demonstrate how miR-122 controlled cell proliferation via the VEGFC/AKT/mTOR signaling cascade, and they suggest that exogenous miR-122 overexpression may be a feasible method for targeted bladder cancer treatments [45]. Guo et al. further investigate the effects of miR-122 on cell proliferation and invasion in bladder cancer. They found that CREB1 was higher in bladder tissues, T24, UM-UC-3, and J82 cells and that miR-122 was increased and inversely linked with cAMP Responsive Element Binding Protein 1 (CREB1). Additionally, CREB1 knockdown reduced the proliferative and invasive abilities of T24 and J82 cells. Furthermore, miR-122 in BC directly targeted and maintained the expression of CREB1. Additionally, they found that in T24 cells, CREB1 may partially counteract miR-122’s effects on cell proliferation and invasion. Thus, recently discovered CREB1 offers a viable therapeutic target for the treatment of bladder cancer and may offer additional insight into the development of the bladder cancer [46].

#### Cervical cancer

One of the most commonly diagnosed tumors in women is cervical cancer (CC) which accounts for most cancer-related deaths. The development of anticancer medicines and the discovery of novel approaches for treating cervical cancer rely on in-depth research of the molecular processes of cervical cancer pathogenesis and the roles of related genes. Yang et al. also made an effort to look into the predictive significance of miR-122 in CC. Their results showed that miR-122, directly targeting RAD21, suppressed cell proliferation and migration while causing apoptosis in cervical cancer. Furthermore, miR-122 was validated to function as a predictive biomarker for patients with cervical cancer, and higher levels of miR-122 expression in CC patients were associated with a better prognosis [47].

### Endocrine system

#### Pancreatic cancer

Among the deadliest cancers is pancreatic cancer (PanC). Although patients may benefit from early diagnosis of the condition, establishing a screening program is challenging due to the low occurrence and the limitations of current pancreatic imaging. Therefore, it is critical to identify innovative therapeutic targets for the treatment of PanC and get a thorough knowledge of the molecular process behind PanC development. In this context, Hu et al. found that miR-122 is sponged by PART1 and promotes pancreatic cancer progression. In this manner, pancreatic cancer patients might benefit from targeting PART1/miR-122 axis as an effective anticancer therapeutic target [48]. According to Yin et al. experimentation, LncRNA SBF2-AS1 stimulated the development of PC cells by functioning like an endogenous RNA competitor to inhibit miR-122-5p and increase XIAP. It was discovered that restricted lncRNA SBF2-AS1 in M2 macrophage-derived exosomes raises miR-122-5p expression to suppress XIAP expression, additional inhibiting PC development [49]. MiR-122-5p targets the CCNG1 gene and inhibits proliferation, migration, and invasion in vitro and its tumor-promoting ability in vivo. Taking into account the results of these studies, miR-122-5p is an increasingly crucial prognostic factor and therapeutic target in pancreatic ductal adenocarcinoma (PDAC) patients [50].

#### Prostate cancer

Worldwide, prostate cancer (PCa) is the second most common cause of cancer death and usually occurs in prostate epithelial cells. In the early stages, prostate cancers typically develop gradually and show no obvious clinical symptoms. As a result, prostate cancer treatment without distant metastases typically has positive effects. Therefore, we must figure out its molecular biology to develop new prognostic markers and therapeutic targets for PCa. Liu et al. investigated the role of miR-122 in prostate cancer. They claimed that in PCa, Rho associated coiled-coil containing protein kinase 2 (ROCK2) expression was increased, and miR-122 expression was decreased. Therefore, low serum levels of miR-122 could be used as a marker for prostate cancer and a sign of poor prognosis. Overexpression of miR-122 was shown to reduce the proliferation of prostate cancer cells and the expression of ROCK2, although treatment with ROCK2 was found to diminish this effect. Consequently, miR-122 may reduce ROCK2 expression, so preventing prostate cancer cells from multiplying [51].

#### Papillary thyroid carcinoma

The most typical endocrine malignancy is papillary thyroid cancer (PTC). It accounts for around 85% of all thyroid tumors with well-differentiated follicular origin. It is categorized as an inactive tumor since its 10-year survival rate is around 93%. To increase the likelihood that PTC patients will be cured and survive, it is crucial to investigate the molecular mechanisms underlying PTC cells’ growth, invasion, and metastasis. By doing this, it will be possible to find compounds that aid in therapy and biomarkers with a changeable expression that may evaluate metastatic progression in PTC. The roles of miR-122-5p in PTC were investigated by Hu et al. The expression pattern of miR-122-5p in PTC cancer tissues and cell lines was examined. The findings showed that PTC cancer tissues, particularly those with substantial invasion or metastasis, had considerably decreased expression of miR-122-5p. The PTC cell line K1 was inhibited in its ability to proliferate, invade, and migrate when miR-122-5p was upregulated due to miR-122-5p mimics but not when miR-122-5p inhibitors were utilized. They additionally verified the miR-122-5p binding sites on the 3'UTR of DUSP4 using a luciferase reporter assay, revealing how miR-122-5p regulates DUSP4 expression specifically. According to these results, miR-122-5p represents a brand-new, highly promising predictive biomarker for PTC [52].

#### Renal cancer

Among fatal urological cancers is renal carcinoma (RC). There are currently no viable treatments available. It is essential to grasp the mechanisms behind RC better to find new biomarkers for RC monitoring and more potent treatments. A recent discovery by Wang et al. demonstrated that upregulated miR-122-5p adversely regulates PKM2, which offers a fresh perspective for advancing RC therapy. This enhances RC cell survival, growth, migration, glycolysis, and autophagy [53]. In addition, Lian et al. demonstrated that miR-122 triggers the PI3K/Akt signaling cascade, which boosts the tendency of renal cell malignancy to invade and migrate [54]. Furthermore, Wang et al. sought to uncover a new molecular target controlled by miR-122 and to ascertain the biological role of miR-122 in RC cancer. When compared to the nearby non-carcinoma tissue samples, they discovered that primary RCC tissue samples had higher levels of miR-122. In addition, both 786-0 and CAKI-1 cells were repressed in migration, invasion, and proliferation by transfection with miR-122 inhibitor. They concluded that miR-122 performs an oncogenic function in renal cancer cell lines, primarily via targeting Sprouty2 [55]. Fan et al. also determined the practical significance and core mechanism of miR-122 in clear-cell renal cell carcinoma (ccRCC) metastasis. According to their findings, ccRCC tissues with metastatic disease exhibited greater miR-122 expression than ccRCC tissues without metastatic disease. It has also been established that increased levels of miR-122 can also be associated with poor metastatic-free survival in patients with localized ccRCC. Additionally, miR-122 downregulated Dicer and its downstream effects, miR-200, by transfection in ccRCC cells, which in turn led to the induction of the EMT [56]. Heinemann et al. investigated the miR-122 biomarker’s potential in ccRCC and demonstrated that elevated blood levels of miR-122-5p signal advanced stage/grade and are indicative of a reduced survival time after nephrectomy for ccRCC. Consequently, miR-122-5p provides a possible serum non-invasive predictive biomarker for patients with ccRCC [57].

### Other cancer

#### Lung cancer

Lung cancer is a complicated condition with numerous histological and molecular subtypes with clinical significance. Since it is the main reason for cancer-related mortality worldwide, it is critical to identify novel diagnostic and prognostic biomarkers. Li et al. looked into sorting miR-122-5p into extracellular vesicles (EVs) and whether EV miR-122-5p was connected to lung tumor progression. They found that the RNA-binding protein hnRNPA2B1 controlled the selective sorting and release of tumor-suppressor miR-122-5p into lung cancer EVs. They found that the RNA-binding protein hnRNPA2B1 was in charge of the tumor suppressor miR-122-5p preferential processing and release into melanoma EVs. In addition, the release of miR-122-5p into EVs was found to have a significant role in advancing lung cancer. Furthermore, they found that EVs produced from lung cancer cells aided liver cell migration by conveying miR-122-5p, a microRNA thought to regulate hepatic metastasis of lung cancer and play a role in pre-metastasis microenvironment development [58]. Additionally, miR-122-5p was found to be down-regulated in non-small cell lung cancer (NSCLC) tissues and to play a tumor suppressor function in the progression of NSCLC, according to Gao’s in vitro and in vivo studies. Additional research identified a binding site for miR-122-5p in the 3ʹ-UTR of Follistatin like 3 (FSTL3), along with a negative correlation between FSTL3 expression and miR-122-5p in samples from NSCLC patients. When miR-122-5p was upregulated, the effect of FSTL3 amplification on the carcinogenic phenotypes of NSCLCs was lessened. MiR-122-5p expression was dysregulated in FSTL3 overexpressed cells, whereas it was elevated in FSTL3 suppression cells. In light of this, miR-122-5p is a valuable new therapeutic target against NSCLC [59]. In addition, Ma et al. looked at how miR-122 affects NSCLC cells, namely A549 cells, to cause radiosensitization. They found that miR-122 makes A549 cells more susceptible to radiosensitization by reducing the expression of cell stress-response regulators including survivin, c-IAP-1, and c-IAP-2. Specifically, they determined that miR-122 inhibits proliferation and radiosensitizes cells by lowering the expression of genes like BCL-W and IGF1R that are essential for survival (i.e., pro-survival or anti-apoptosis). In general, their findings indicate that miR-122 increased the NSCL C cell line’s sensitivity to radiation, and miR-122 expression using an adenoviral vector may represent a potential approach for NSCLC irradiation or gene therapy [60]. Chandimali et al. examined Prx II expression and structural relationships explaining the capability of Prx II in promoting cancer stem cells (CSCs) features including stemness, cell proliferation, metastasis, and angiography in gefitinib-resistant A549 (A549/GR) stem cells. Their findings demonstrated that miR-122-transfected cells had lower Prx II expression than miR-NC (control)-transfected cells, indicating that miR-122 overexpression suppressed Prx II in A549/GR stem cells. They also found that suppressing Prx II expression with miR-122 prevented the stem cell features induced by greater Prx II expression in A549/GR stem cells, such as cell proliferation, migration, invasion, EMT, self-renewal, and angiogenesis. They further revealed that downregulating the Hedgehog, Notch, and Wnt/-catenin cascades is the primary mechanism by which miR-122’s anti-stemness function is regulated. As a result, increasing miR-122 expression in A549/GR stem cells may be useful in the treatment of NSCLCs [61].

#### Osteosarcoma

Osteosarcoma (OS) is deadly cancer that mostly affects children and teenagers. Discovering innovative strategies to increase the survival and prognosis of osteosarcoma patients requires understanding the molecular mechanisms underlying the disease. This can be done through screening molecular biomarkers and gene-targeted medicinal medicines. It was postulated by Ma et al. that a LINC01410/miR-122-5p/NDRG3 axis contributed to OS development and influenced the proliferation and invasion of OS cells. MiR-122-5p expression was significantly suppressed in OS, according to their research. They further found that LINC01410 interacts with miR-122-5p, and miR-122-5p interacts with NDRG3. Cells of OS were found to be inhibited in proliferation, invasion, and migration by knocking down LINC01410. MiR-122-5p was upregulated, while NDRG3 was downregulated due to LINC01410 being knocked down. As a result, the LINC01410/miR-122-5p/NDRG3 axis is engaged in OS development [62]. Furthermore, Li et al. investigated the role of the TP53 gene targeting miR-122 in mediating the PI3K-Akt-mTOR signaling pathway on the OS cells proliferation and apoptosis. They discovered that boosting miR-122-5p has effects similar to those of upregulating TP53, including a reduction in osteosarcoma cell proliferation and an increase in apoptosis. This is achieved by blocking PI3K-Akt-mTOR signaling. They reported that TP53 up-regulation by miR-122-5p overexpression may reduce the proliferation and enhance the apoptosis of osteosarcoma cells by suppressing the activation of the PI3K-Akt-mTOR signaling pathway [63]. In addition, an experimental investigation by Yuankey et al. disclosed that ADAM metallopeptidase domain 10 (ADAM10) promotes osteosarcoma cell proliferation, migration, and invasion by regulating E-cadherin/β-catenin signaling pathway and miR-122-5p can target ADAM10, indicating that miR-122-5p/ADAM10 axis might serve as a therapeutic target of osteosarcoma [64].

#### Glioma

Although malignant gliomas are the most frequent kind of intracranial cancer in humans, they have a dismal survival rate because of their high recurrence and invasiveness rates [65]. However, their malignant features and the molecular processes behind them remain poorly understood [66]. Sun et al. confirmed the complementary binding between the lncRNAs urothelial carcinoma-associated 1 (UCA1) and miR-122 at the 3′-UTR using bioinformatics analysis and luciferase reporter experiment. They also demonstrated through functional assays that UCA1 worked as a “sponge” for miR-122 to control the migration, invasion, and proliferation of glioma cells [67]. Wang et al. also investigated the effect of miR-122 in glioblastoma processes. They discovered that miR-122 and the Wnt/β-catenin pathway form a regulatory network in glioma cells. MiR-122 reduced WNT1 protein expression. But it was discovered that the Wnt β-catenin pathway negatively regulates the miR-122 transcript. MiR-122 is a potential therapeutic target for the treatment of gliomas and contributes to their progression [68].

#### Hematological malignancy

Since the median age for most of these ailments is between 65 and 70, hematologic malignancies are typically diseases of the elderly. Hematological malignancies are diagnosed chiefly based on clinical presentation, morphology, histology, cytogenetic, immune-phenotyping, and molecular genetic data. Still, it is frequently unsuccessful in identifying the molecular route that is responsible for carcinogenesis. Consequently, the discovery of novel biomarkers for early diagnosis and prognosis, as well as research into those biomarkers, is vital for improved cancer control. The research conducted by Yang et al. examined the clinical importance of miR-122 and its roles in malignant phenotypes of childhood acute myeloid leukemia (AML). They discovered that forced expression of miR-122 effectively slowed cell growth and decreased the proportion of S-phase cells in AML cell lines. They found that aberrant expression of miR-122 may indicate a more aggressive course in childhood AML. Notably, its downregulation might operate as a prognostic indicator for bad results. Additionally, their research suggests that miR-122 may act as a tumor suppressor in children with AML, revealing a potential new treatment option for this cancer [69]. Beg et al. also sought to assess the clinical significance of miR-122 expression concerning imatinib response in chronic myelogenous leukemia (CML) patients. They conclude that imatinib treatment enhances the expression of miR-122 in CML patients and that miR-126 identifies those who respond well to imatinib therapy [70].

#### Melanoma

One of the tumors with the quickest rate of growth in the globe is malignant melanoma. Surgery can treat initial cutaneous melanomas; however, advanced, metastatic melanomas cannot be treated with surgery alone and demand superior therapeutic strategies. More profound knowledge of the molecular etiology of malignant melanoma is highly required in light of the high mortality rates brought on by metastatic melanoma. Li et al. looked at melanoma cell lines SK-MEL-110 and A375 to see how miR-122-5p affected proliferation, cell cycle progression, and apoptosis. A higher level of miR-122-5p expression was seen in melanoma tissues, suggesting a role for this miRNA in the progression. Their experiments revealed that miR-122-5p inhibitor significantly slowed the growth of SK-MEL-110 and A-375 cells and increased the proportion of cells in the G1 phase of the cell cycle but had no discernible effect on apoptosis. Additionally, the miR-122-5p inhibitors boosted the expression of NOP14 protein but had little effect on the quantity of NOP14 mRNA. Thus, miR-122-5p prevents SK-MEL-110 and A-375 cells from proliferating, possibly by disrupting the cycle via NOP14 [71].

#### Oral squamous cell carcinoma

**Fig. 2:**
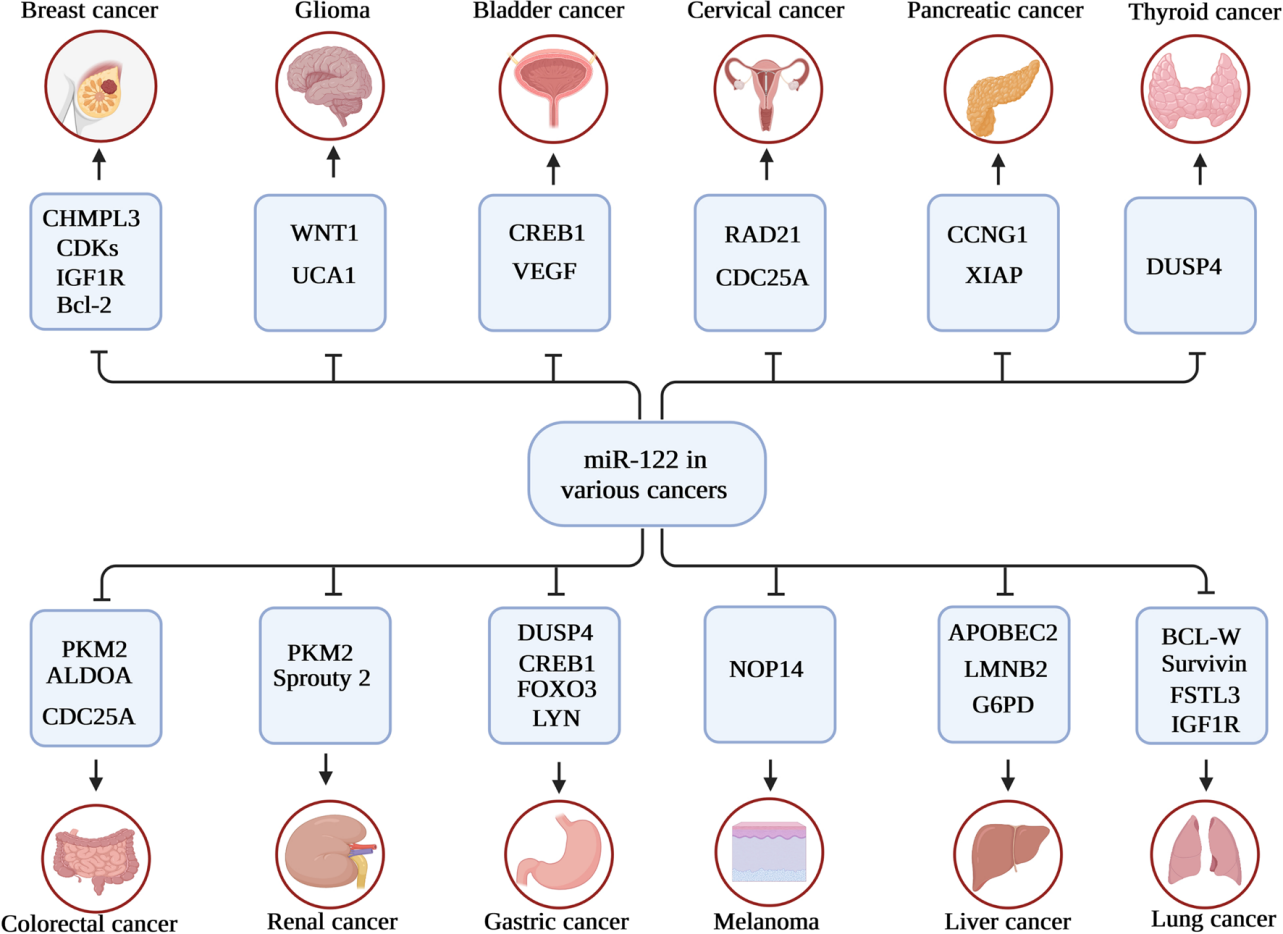
miR-122 target different mRNA in different tissues

A diverse category of malignancies called oral squamous cell carcinomas (OSCC) develops from the mucosal layer of the mouth cavity. Its prevalence has increased by 50% during the previous decade. Almost all cases of cancer in the head and neck are this kind. Advances in the early detection and treatment of OSCC are being made possible by a more excellent knowledge of the molecular changes contributing to OSCC development. The LINC01410/miR-122-5p/NDRG3 axis was explored by Tian et al. to determine its function in the development of OSCC. It was found that LINC00974 was negatively correlated with miR-122 in OSCC tissues but positively related to RhoA. Additionally, RhoA was elevated by LINC00974 overexpression, while RhoA was dysregulated by miR-122 overexpression. Furthermore, LINC00974 overexpression downregulated miR-122, while miR-122 overexpression had little effect on LINC00974 expression. When cancer cells were tested for invasion and migration, miR-122 overexpression lowered the rate of invasion and migration, but LINC00974 and RhoA overexpression boosted it. In addition, LINC00974 overexpression was mitigated by miR-122 overexpression. Thus, in OSCC, LINC00974 can increase the expression RhoA and downregulate miR-122, which encourages cell migration and invasion [72] (Fig. 2).

## Prognosis, and diagnosis perspective of miR-122 in human cancer

Delayed cancer diagnosis and inefficient cancer prognosis determination are problems faced in cancer diagnosis and treatment. MiRs, especially miR-122, have shown a promise in cancer diagnosis and prognosis. In this context, in a large and well-characterized sample of CCA patients receiving tumor excision, Loosen et al. also sought to assess the diagnostic and prognostic utility of miR-122. Their research revealed that miR-122 analysis offers a promising method for the diagnosis of CCA, even in its earliest stages, and that a post-operative decrease in miR-122 serum concentrations may be a sign of a positive outcome and valuable in identifying patients who will recover well from extensive liver surgery [73]. Beside, Mazza et al. sought to discover new miRNAs in plasma that might be used to distinguish between PanC and healthy individuals (HS) in comparison to CA19-9, as well as to forecast the clinical phenotypes and fates of the patients. They concluded that higher plasma miR-122-5p is associated with more adverse clinical outcomes and prognosis [74]. Notably, the expression of miR-122 and its role in glioma diagnosis and prognosis was studied by Tang et al. They discovered that miR-122 expression was dramatically downregulated in the plasma of glioma patients compared to healthy people and that low miR-122 expression was associated with a poor prognosis. As a result, miR-122 may function as a standalone prognostic factor to predict a poor glioma prognosis [75]. Importantly, the expression of miR-122 in LC patients before and after chemotherapy has been quantified by Zhan et al. and their prognostic significance was assessed. They discovered lower miR-122 expression levels in LC tissues, demonstrating their ability to identify malignant tumors from surrounding tissues. They also discovered that sorafenib chemotherapy boosted its levels in the peripheral blood and that miR-122 predicted how well sorafenib chemotherapy worked. Next, the researchers observed that low levels of miR-122 were linked to decreased 5-year survival rates and were independent risk factors for shorter survival in LC [76]. Furthermore, Akuta et al. through the long-term analysis of successive liver biopsies, found that miR-122 circulation dynamics in Japanese individuals with histopathologically diagnosed NAFLD and severe fibrosis stage could predict liver cancer and mortality [77]. Moreover, in order to determine whether plasma levels of particular miRNAs are related to distant metastasis (DM) in gastric cancer, Chen et al*.* conducted an extensive research study. Using miRNA microarray screenings and additional qRT-PCR confirmation, they discovered that miR-122 expression was considerably lower in patients with gastric cancer with distant metastases (GC/DM) compared to GC/NDM, and greater plasma levels of miR-122 in GC predict a favorable prognosis. This suggests that a reduction in circulating miR-122 may aid in the early diagnosis of DM in GC [78]. Similarly, S Maruyama et al. looked into the clinical use of AFPGC-specific miRNA for patient monitoring and prognostication. Their results unmistakably showed that AFPGC tissues had much higher levels of miR-122-5p expression than normal and non-AFPGC tissues. Additionally, compared to plasma samples from healthy volunteers and patients without AFPGC, patients with AFPGC had greater expression levels of this miRNA, which was somewhat linked with plasma AFP levels. MiR-122-5p may be helpful as a prognostic biomarker, particularly for liver metastasis in this small GC subgroup, because tissue expression levels of the gene showed a greater connection with malignant potential than plasma AFP levels in AFPGC patients [79]. In addition, Maierthaler et al. investigated the potential predictive usefulness of circulating miR-122 for CRC. They discovered that miR-122 might be particularly useful for predicting metastasis and recurrence in non-metastatic CRC patients and tracking their therapy (especially liver metastasis). Additionally, modifying the course of treatment based on the prognosis may increase the likelihood that the patients will survive [80]. Therefore, we hypothesize that miR-122 can also be used as a prognostic biomarker in addition to its diagnostic role.

## MiR-122 in cancer therapy resistance

**Fig. 3:**
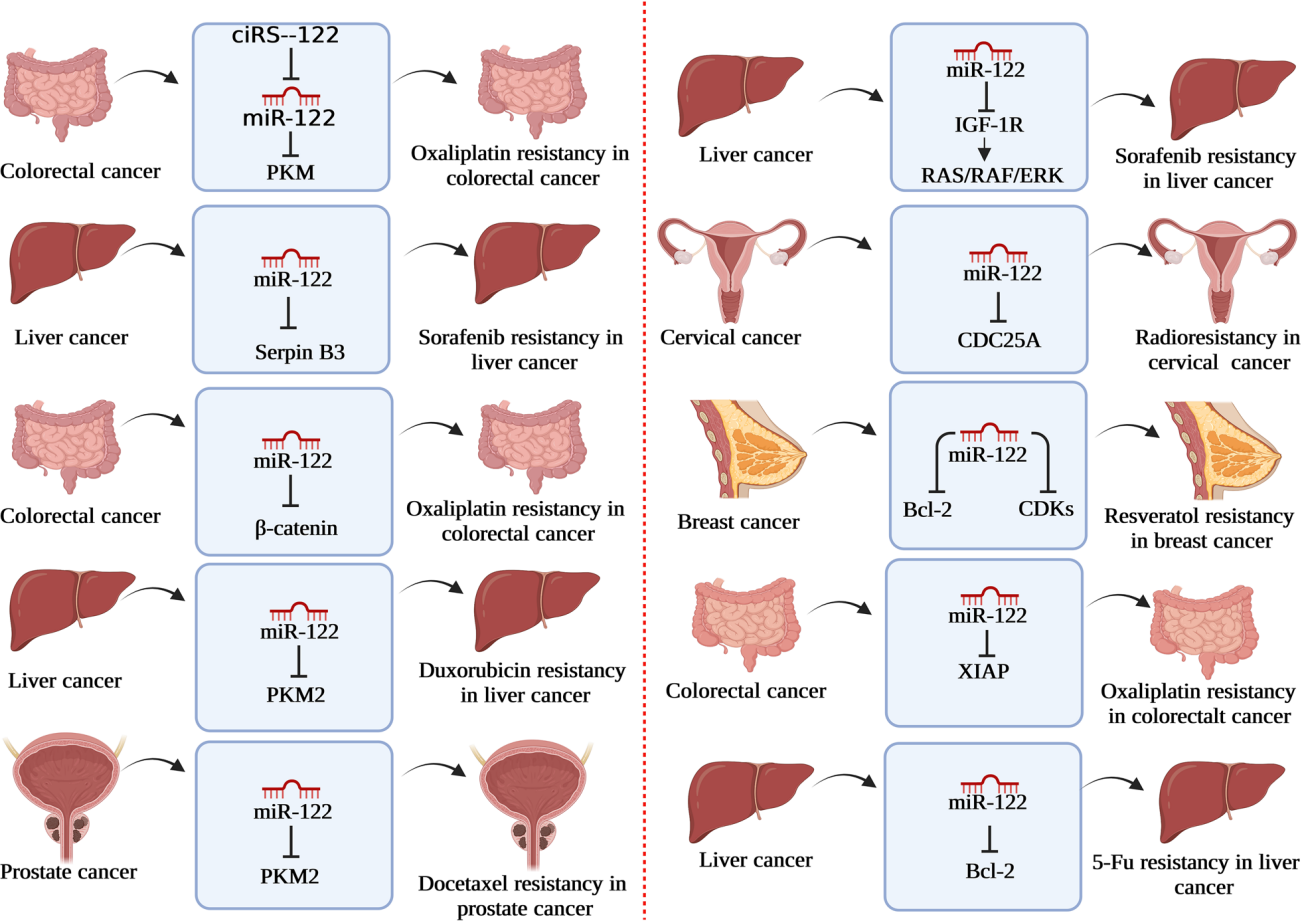
An overview of the regulatory role of miR-122 in cancer therapy resistance in different cancers*.* Recent studies have shown that miR-122 by targeting different proteins plays a major role in resistance to different cancer therapies

Resistance to tumor therapy is challenging in cancer treatment due to the daunting range of resistance mechanisms. Cumulative evidence showed that miR-122 was correlated with chemotherapy resistance, suggesting that it might serve as a candidate and promising biomarker for drug resistance. This will be presented in the following section (Fig. 3).

Sorafenib resistance: Turato et al. examined how miR-122 and SerpinB3 affected HCC cell phenotype and sorafenib resistance. Their luciferase experiment and bioinformatics analysis showed that SerpinB3 mRNA and miR-122 interact. In addition, transfection of miR-122 reduced SerpinB3 mRNA and protein levels in HepG2 cells that overexpressed SerpinB3, and suppression of miR-122 boosted SerpinB3 expression. They also found that overexpressing miR-122 enhanced susceptibility to sorafenib in treated cells but not in cells overexpressing SerpinB3. Finally, they found that anti-miR-122 transfection improved cell viability in sorafenib-treated Huh-7 cells. Their research led them to conclude that miR-122 binds to SerpinB3 and that low levels of miR-122 are linked to SerpinB3 positive and a stem-like phenotype in HCC. In this manner, miR-122 replacement treatment with sorafenib merits consideration as a therapeutic option in SerpinB3-negative HCCs [81]. Sorafenib resistance in HCC cells was also studied by Xu et al. Expression of the liver-specific miR-122 was shown to be considerably lower in sorafenib-resistant cells, as determined by miRNA microarray analysis. They found that drug-resistant cells could be rendered responsive to sorafenib and caused apoptosis with overexpression of miR-122. In addition, they demonstrated that the Insulin-like growth factor 1 receptor (IGF-1R) is a target of miR-122 and that activation of IGF-1R by IGFI or IGFII inhibited miR-122-induced apoptosis. Their results demonstrated that the activation of IGF-1R caused sorafenib resistance through ectopic down-regulation of miR-122, which counteracted the drug’s tendency to induce apoptosis. Their follow-up experiments showed that down-regulation of miR-122 led to IGF-1R activation, leading to RAS/RAF/ERK signaling activation, all of which were linked to drug resistance. Thus, abnormal miR-122 and IGF-1R expression significantly determine sorafenib tolerance [82].

Oxaliplatin resistance: Wang et al. looked into the causes of colorectal cancer’s medication resistance. In vitro and in vivo experiments demonstrated that oxaliplatin-resistant cell exosomes transported circular RNA hsa_circ_0005963 (termed ciRS-122) to susceptible cells, where it stimulated glycolysis and drug resistance by miR-122 sponging and upregulating PKM2. Furthermore, exosomal si-ciRS-122 was demonstrated to be effective in vivo at decreasing glycolysis and reversing oxaliplatin resistance by regulating the ciRS-122-miR-122-PKM2 pathway [83]. Furthermore, Hua et al. investigated how miR-122 affected CRC’s ability to become oxaliplatin-resistant. They found that an X-linked inhibitor of apoptosis protein (XIAP) was expressed at significantly higher levels in oxaliplatin-resistant SW480 and HT29 cells (SW480/OR and HT29/OR) compared with normal SW480 and HT29 cells and that miR-122 expression was significantly lower in SW480/OR and HT29/OR. Overexpression of XIAP was shown to contribute to oxaliplatin resistance in CRC cells, and the authors showed that this was partly due to the downregulation of miR-122. They followed this by discovering that restoring miR-122 expression in SW480/OR and HT29/OR cells might make them more vulnerable to apoptosis induced by oxaliplatin by suppressing XIAP expression. Thereby, miR-122 reduced oxaliplatin resistance in CRC by targeting XIAP, leading the researchers to infer that XIAP overexpression in CRC cells is to blame for the development of acquired resistance to the oxaliplatin [84]. Moreover, Cao et al. investigated miR-122’s role in HCC’s oxaliplatin resistance. They discovered that miR-122 was decreased in HCC cells but that up-regulating miR-122 or inhibiting Wnt/-catenin signaling boosted apoptosis and made HCC cells more sensitive to OXA. Furthermore, their molecular analysis showed that miR-122 targeted and suppressed the Wnt/-catenin pathway, while β-catenin linked to the MDR1 promoter and boosted its transcription. Thereby, through downregulation of the Wnt/-catenin pathway, miR-122 reduces MDR1 expression and makes HCC more sensitive to OXA. As a result, miR-122 represents an exciting new therapeutic target for the treatment of HCC [85].

Radioresistance: Perez-Aorve et al. investigated the critical functions that miR-122 plays in radioresistant breast cancer and the processes underneath its function. They discovered 16 deregulated miRNAs using differential expression profiling assays in obtained radioresistant breast cancer cells, among which miR-122 was found to be increased. Functional investigation revealed that miR-122 is upregulated in radioresistant breast cancer to enhance cell survival and that miR-122 controls responses to radiation in a cell-phenotype-dependent way, serving as both a repressor and an oncomiR [86]. Moreover, miR-122-5p and CDC25A were examined by Ding et al. to see how they were expressed in cervical cancer cells and how they affected the cells' radiosensitivity. In cervical cancer tissues and cells, they revealed that miR-122-5p is poorly expressed while CDC25A is substantially expressed. They established CDC25A as a target of miR-122-5p using the TargetScan database and in vitro studies, including a dual-luciferase reporter test. Furthermore, X-ray radiation has been demonstrated to increase CDC25A’s expression, increasing radiation resistance in cervical cancer cells. However, overexpression of miR-122-5p or suppression of CDC25A renders cervical cancer colonies unable to survive and causes them to undergo apoptosis. Consequently, miR-122-5p targets CDC25A, causing cervical cancer cells to become more radiosensitive [87].

Resveratrol resistance: Zhang et al. designed experiments to investigate the functional role of resveratrol in MCF-7 cells (low-invasive breast cancer) concerning Adriamycin chemosensitivity and identify the mRNAs that resveratrol targets and the essential proteins that are involved in cellular activity. They discovered that by manipulating the critical suppressor miR-122-5p, adriamycin-resistant breast cancer cells were sensitive to resveratrol’s chemotherapeutic effects, with cell cycle arrest and apoptosis being the most notable effects. Additional miRNA manipulation using miR-122-5p mimics or inhibitors revealed that miR-122-5p significantly affected the regulation of critical antiapoptotic proteins (B-cell lymphoma 2 [Bcl-2]) and cyclin-dependent kinases (CDK2, CDK4, and CDK6) in resveratrol-resistant breast cancer cells. Therefore, miR-122-5p targets Bcl-2 and CDKs in breast cancer cells, which assists in cell-cycle arrest [88].

Doxorubicin (DOX): Doxorubicin (DOX) resistance in hepatocellular cancer cells was investigated by Pan et al. In contrast to parental Huh7 cells, doxorubicin-resistant Huh7 (Huh7/R) cells showed a down-regulation of miR-122, indicating miR-122 is involved in the chemoresistance. Using a luciferase reporter assay, they demonstrated that PKM2 is the direct target of miR-122 and that glucose metabolism is dramatically increased in Huh7/R cells. Their follow-up investigations revealed that miR-122 overexpression in Huh7/R cells restored doxorubicin resistance via inhibiting PKM2, resulting in the doxorubicin-resistant cancer cells' apoptosis. They concluded that doxorubicin resistance is associated with aberrant glucose metabolism and that miR-122-induced suppression of glycolysis may be a functional therapeutic approach for treating doxorubicin-resistant hepatocellular cancer [89].

Docetaxel resistance: Zhu et al. looked into the function of miR-122 in the chemoresistance of PCa cells and the possible mechanisms. Their findings showed that miR-122 could reverse the resistance of LNCaP/Docetaxel cells to docetaxel and that miR-122 overexpression might increase docetaxel-induced apoptosis in PCa cells. This may be due to miR-122’s ability to regulate its target protein PKM2 by binding to the 3'-UTR. These results establish a causal relationship between miRNAs and PCa chemoresistance, suggesting that miRNA-122 targeting may represent a unique therapeutic approach for PCa chemoresistance [90].

## MiR-122 and cancer stem cell

**Fig. 4:**
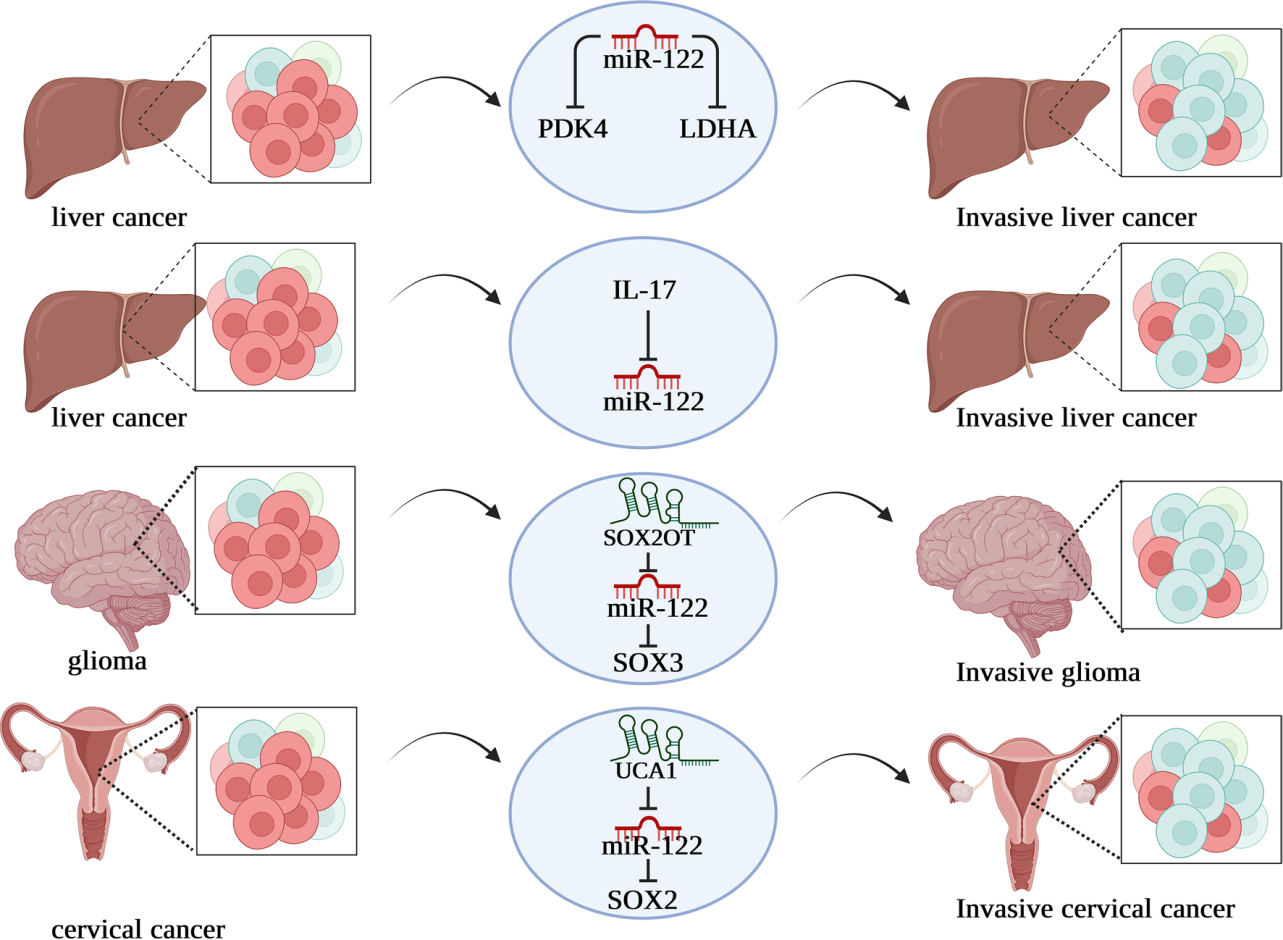
An overview of the regulatory role of miR-122 in cancer stem cells in different cancers. Recent studies demonstrated that miR-122 by targeting various proteins plays a crucial in the stemness of cancer stem cells

Numerous cancers include a small population of cancer stem cells, cells able to self-renew and differentiate. Inappropriate control of stem cell self-renewal is necessary for the emergence and development of cancer. In addition, the presence of cancer stem cells is likely responsible for resistance to standard cancer therapies and recurrence in patients. A better knowledge of the biology and processes linked with cancer stem cells may lead to better cancer therapies and, maybe, a cure. There is mounting evidence that miRNAs have a role in deregulating cancer stem cells. Recent research suggests that miR-122 plays an essential role in cancer stem cell activity by regulating several proteins (Fig. 4). In this context, Song et al. sought to identify the functional relevance of miR-122 in CD133 ( +) liver CSCs and CD133 (−) cells. Their findings demonstrate that CD133 ( +) hepatocellular CSCs differ from CD133 (−) cells in their metabolic profile, with the former preferring glycolysis over oxidative phosphorylation. Their results showed that blocking Pyruvate Dehydrogenase Kinase 4 (PDK4) and Lactate dehydrogenase A (LDHA) significantly reduced CD133 + stemness features and helped patients overcome sorafenib resistance (chemotherapy medication currently used for hepatocellular cancer). They also showed that CSC features, such as an increase in the CD133 ( +) cell population, stemness gene expression, and spheroid formation, are induced in CD133 (−) cells by the glucose and lactate addition. Through targeting PDK4, their active investigations demonstrated that miR-122, which is abundantly expressed in the liver, inhibits CSC features. According to their claims, enhanced glycolysis is associated with CD133 ( +) stem-like features, and metabolic reprogramming with miR-122 or PDK4 may be a cutting-edge treatment option for hepatocellular carcinoma [91]. Similarly, Gasmi et al. proposed that miR-122 promotes LPC transition into a CSC phenotype, which may play a role at the beginning of liver cancer. Their study provided in vitro proof that long-term IL-17 activation of LPCs caused them to develop into CSCs. Indeed, they gained the ability to express CSC markers and to engage in self-renewal, as shown by an increase in their capacity to form spheroids. Using miRNome analysis, they determined that following chronic treatment with IL-17, miR-122 expression in LPCs decreased by 90%. In an immunodeficient mouse model, they discovered that ectopic engraftment of LPCs in an IL-17-enriched environment triggered tumor formation with an aggressive character. As a result, LPCs are more likely to develop into CSCs after exposure to the cytokine IL-17 over an extended period. Thus, methods that target IL-17 reduce CSC incidence and liver tumor development via miR-122 restoration [92]. Furthermore, Gao et al. investigated the role of exosomal lncRNA urothelial cancer-associated 1 (UCA1) in modulating SOX2 expression in CC stem cells (CD133 + CaSki) by sponging miR-122-5p. CC stem cells (CD133 + CaSki) were discovered to have increased UCA1 and SOX2 and decreased levels of miR-122-5p. Exosomes were discovered to inhibit apoptosis in CD133 + CaSki cells while encouraging migration, invasion, and proliferation. Subsequent functional experiments showed that inhibiting UCA1 or increasing miR-122-5p degraded SOX2 expression, lowered invasion, migration, and proliferation of CD133 + CaSki cells, accelerated apoptosis, and decreased the tumor volume and weight in nude mice. Thus, their findings imply that exosomes transporting UCA1 as a ceRNA of miR-122-5p enhance the expression of SOX2, thus influencing CC stem cell self-renewal and differentiation [93]. The role of lncRNA-SOX2OT and potential molecular pathways by which it affects the biological behaviors of glioblastoma stem cells (GSCs) were further clarified by Su et al. They used real-time PCR to show that SOX2OT expression was elevated in glioma tissues and GSCs. They found that knocking down SOX2OT reduced GSC proliferation, migration, and invasion while increasing their susceptibility to apoptosis. MiR-122 was shown to be down-regulated in both human glioma tissues and GSCs, and it was found to exhibit tumor-suppressive effects by reducing GSC proliferation, motility, and invasion and increasing their susceptibility to apoptosis. Using a dual-luciferase reporter assay, they discovered that SOX2OT is linked to miR-122, and they also discovered that knocking down SOX2OT significantly upregulated miR-122 expression in GSCs. SOX3 and TDGF-1 were both upregulated in human glioma tissues and GSCs. Furthermore, knocking down SOX3 impeded GSC growth, migration, and invasion, increased GSC mortality, and reduced TDGF-1 expression by interacting directly with the TDGF-1 promoter. MiR-122 over-expression reduced SOX3 expression by targeting its 3'UTR, whereas reduction of SOX3 suppressed SOX2OT expression by binding to the SOX2OT promoter directly, creating a positive feedback loop. Notably, reduced proliferation, migration, and invasion of GSCs, increased apoptosis in GSCs, and decreased activity in the JAK/STAT signaling pathway were all significant results of TDGF-1 knockdown. As a result, the SOX2OT-miR-122-SOX3-TDGF-1 pathway establishes a positive feedback loop and governs the biological activities of GSCs; this knowledge may lead to a new approach to treating gliomas [94]. Therefore, correcting miR-122 dysregulation in cancer stem cells with genetic techniques may avert invasive malignancy.

### Mir-122-mediated therapy

MiRNAs have recently been suggested as prospective therapeutic candidates as cancer therapy [95]. Tumor repressor miRNAs are a promising candidate for developing cancer treatments due to their regulatory capacity, which permits modulation of whole signaling pathways inside the cells. As a result, over the past few decades, scientists have pursued the development of novel approaches that would powerfully and safely restore these suppressive miRNAs in cancerous cells powerfully and safely. As a result, the sections below have explored numerous methods to raise microRNA amounts in malignancies.

*Treatment mediated by exosome:* Exosomes are tiny membrane vesicles that are released by the majority of cell types. They are common in bodily fluids and cellular supernatants. Exosomes transfer their contents, containing proteins, lipids, and RNAs, across cells, and there is mounting indication that they are crucial for intercellular interaction on both a localized and systemic level [96]. The ability of cell-derived exosomes to get past biological barriers and allow the inserted gene and medicine to access the desired tissue, which has been extremely difficult for synthetic carriers, is of particular importance for regulated pharmaceutical delivery. The development of exosome-based pharmaceutical-delivering methods for treating cancer has received much scientific attention [97]. To find out if Mesenchymal stem cells (MSC) exosomes produced from adipose tissue (AMSC) may be employed to deliver miR-122 in this situation, Lou et al. did an investigation. According to their research, miR-122-transfected AMSC can efficiently package miR-122 into exomes that later will be released, which can regulate miR-122 communication between AMSCs and HCC cells. This makes cancer cells more susceptible to chemo-drugs by changing the expression of miR-122-target genes in HCC cells. Additionally, intra-tumor administration of 122-Exo markedly improved the anticancer effectiveness of sorafenib in HCC in vivo [98]. Additionally, Jiao et al. looked into the role of exosomal miR-122-5p in the development of gastric cancer. They discovered that exosomal miR-122-5p, which targets GIT1, significantly reduced GC cell growth, motility, and penetration in vitro and tumor formation in vivo [99]. Exosomal miRNAs isolated from the ovaries of patients with a primary ovarian deficiency were studied for their clinical impact and probable mechanisms by Zhang et al. They recovered exosomes from ovarian tissues, cultured ovarian granulosa cells (GCs), and ovarian tissues and found variably expressed miRNAs in the ovarian exosomes of mice treated with CTX. Afterward, they demonstrated how healthy ovarian exosomes maintain follicles, and they discovered that miR-122-5p suppressor shielded ovarian GCs from CTX-induced death by targeting the gene BCL9 [100].

*Treatment mediated by nanoparticels*: Lately, nanoparticles have emerged as a desirable method for miRNA transport to tumors [101]. Various nanoparticle compositions have been used to accomplish this, involving bacterial minicells made by genetic engineering, silica, gold, and polyamidoamine (PAMAM) dendrimers, which are generated synthetically to construct nanoscale delivering vehicles [102]. Sendi et al. describe the creation of a pharmaceutical drug using a nano-formulated miR-122 to stop liver metastasis. They created a nano-formulated miR-122, a galactose-targeted lipid calcium phosphate (Gal-LCP). Their research found that miR-122 transmission was linked to the downregulation of essential genes implicated in the inflammatory routes that lead to cancer metastasis, namely several pro-inflammatory factors, matrix metalloproteinases, and other extracellular matrix breakdown enzymes. The liver is more receptive to an antitumor immunological reaction as a result of Gal-LCP miR-122 therapy because of its enhanced CD8 + /CD4 + T-cell ratio and reduced cell-infiltrated immunosuppression [103]. Additionally, perfluoropentane/C9F17-PAsp(DET)/miR-122/poly(glutamic acid)-g-MeO-poly(ethylene glycol) (PGA-g-mPEG) ternary nanodroplets (PFP-TNDs/miR-122), were created by Guo et al. to transfer miR-122 for the treatments of HCC. They discovered that PFP-TNDs/miR-122, in combination with ultrasound waves, efficiently reduced HCC cell growth, migration, and invasion while also preventing tumor formation in mice [104]. In addition, Xiong et al. developed a cyclodextrin-cored star copolymer nanoparticle system (sCDP/DOX/miR-122) to transport miR-122 and doxorubicin (DOX) together for the treatment of hepatoma. They discovered that sCDP/DOX/miR-122 efficiently transported DOX and miR-122 into hepatoma cells and that miR-122 can subsequently be explicitly released from the nanosystem, increasing chemosensitivity and having synergistic consequences on cell proliferation inhibition [105]. Additionally, Yuan et al. created graphene nanocomposites (GGMPN) containing gold nanoparticles packed with miR-122 and monoclonal P-glycoprotein (P-gp) antibodies, which enhanced drug-Ft HepG2 cell death with therapeutic targeting and managed to release capabilities [106].

**Fig. 5:**
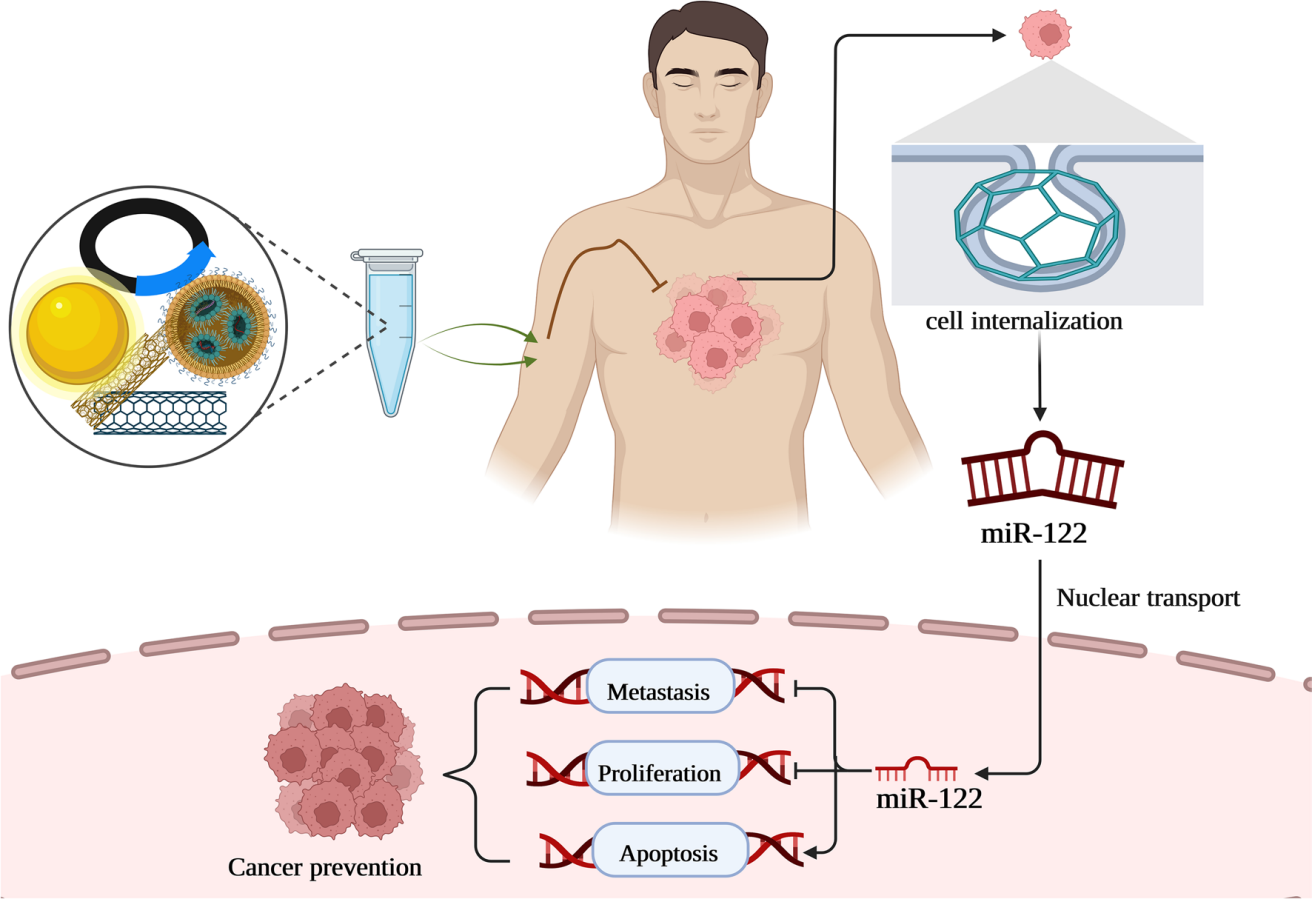
miR-122 replacement therapy via various tools to combat cancer

*Treatment mediated by viral vector*: Zhou et al. created an adenoviral vector carrying the miR-122 MRE to limit TRAIL expression in esophagus cancer cells and stop TRAIL action in liver tissues. They discovered proof that miR-122 prevented liver damage brought on by TRAIL and inhibited the growth of esophagus cancer xenografts. Ad-TRAIL-122 can consequently effectively shield liver tissue from damage by adenoviruses that produce TRAIL [107]. Additionally, Qin et al. sought to describe the possible role and mechanisms of miR-122 in the therapy of lung cancer. They showed that miR-122 expression by an adenoviral vector had potent anticancer effects and successfully stopped NSCLC cells from spreading. Additionally, miR-122 slowed down the EMT process in NSCLC cells [108]. In addition, Ma et al. revealed a brand-new approach to cancer treatment relying on adenoviral vector-based miR-122 gene transfer. Infection of tumor cells with Ad-miR122 prevented the proliferation of cancer cells from the liver (HepG2, Hep3B, Huh7, and PLC/PRF/5), the lung (NCI-H460), and the uterine cervix, according to their research. Their additional investigation revealed that anticancer action was connected to the cell cycle interruption and/or production of apoptosis in carcinoma cells [109]. Additionally, AAV3-miR-26a/122 carriers were tested by Yin et al. for their ability to inhibit the formation of human liver cancers in mice and HCC cells in vitro. In a mouse xenograft model, they found that cAAV3-miR-122 vectors caused a 70% reduction in the development of Huh-derived human liver cancers [110] (Fig. 5).

### Conclusion

When it comes to controlling the production of proteins, miRNAs perform a wide variety of regulatory tasks. It has been demonstrated that aberrant miRNA expression aids in cancer initiation and/or advancement. Findings from cancer cell lines revealed that miR-122 functions as an oncogenic or tumor-suppressive miRNA and examination of patient tumor samples furthermore supported the idea that miR-122 dysregulation is a crucial component in the growth of malignancies. Furthermore, analysis of the levels of miR-122 expression in various tissues showed that the physiologic function of miR-122 in carcinoma seemed to vary on the kind of cancer (Table 1). These all pointed to the use of miR-122 as a cutting-edge tool for cancer treatment. The miRNA replacement provides a novel pharmacological idea. It seeks to recover lost function, which is generally unreachable for medicines designers. Additionally, it expands on the notion that effective cancer treatments depend on the modulation of numerous biological networks linked to human disease. Data from animals and cells indicate that pharmaceutical release of miRNA mimics efficiently slows the progression of tumors while being well handled by healthy tissues. These results highlight the route to a miR-122 treatment in the clinic.

**Table 1:** miR-122 expression in human cancer

Cancer	Author/year	Verified target	Remark	Expression levels	Sample	miR-122/lncRNA or circular RNA axis	Tumor suppressor/Enhancer	Refs.
Liver	Barajas et al./2018	G6PD	Modulate glucose metabolism in liver cancer	↓	Cell lines	–	Suppressor	[18]
	Li et al./2020	LMNB2	Inhibit hepatocarcinoma progression	↓	Tissues samples/cell lines	–	Suppressor	[19]
	Li et al./2019	APOBEC2	Involved in HBV infection	↓	Cell lines	–	Suppressor	[21]
	Cheng et al./2018	–	Blocked tumorigenicity	↓	Tissues samples/cell lines	lncRNA-HOTAIR	Suppressor	[22]
	Lee et al./2021	–	Increased serum levels of miR-122-5p associated with increased risk of incident LC in T2D	**↑**	Serum samples	–	Enhancer	[23]
	Zhan et al./2021	–	Can predict LC patient responses to sorafenib chemotherapy	↓	Tissues samples	–	Suppressor	[76]
	Yin et al./2016	TGFβ1	Exerts markedly different effects on metastatic liver cancer in humans and mice	↓	Cell lines	–	Suppressor	[111]
	Shyu et al./2016	PEG10	Serve as early biomarkers for identifying an HCC subpopulation that is at high risk for poor outcome	↓	Tissues samples/cell lines	–	Suppressor	[112]
	Akuta et al./2022	–	Predicting LC for NAFLD patients with severe fibrosis stage	↓	Serum samples	–		[77]
	Coulouarn et al./2009		An important determinant in the control of cell migration and invasion	↓	Tissues samples/cell lines	–	Suppressor	[113]
Gastric	Meng et al./2020	LYN	Inhibited the proliferation, migration, and invasion	↓	Tissues samples/cell lines	–	Suppressor	[24]
	Xu et al./2018	DUSP4	Restrained migration and invasion	↓	Tissues samples/cell lines	–	Suppressor	[25]
	Rao et al./2017	CREB1	Inhibited GC tumorigenesis	↓	Tissues samples/cell lines	–	Suppressor	[26]
	Pei et al./2017	MYC	Inhibits tumor cell proliferation and induces apoptosis	↓	Cell lines	–	Suppressor	[27]
	Chen et al./2014	–	Function as potential novel biomarkers for the early detection of distant metastasis	↓	Cell lines/plasma samples	–	Suppressor	[78]
	Maruyama et al./2019	FOXO3	Inhibited apoptosis	**↑**	Tissues samples/cell lines	–	Enhancer	[28]
	Maruyama et al./2018	–	Useful biomarker for early detection and disease monitoring	**↑**	Tissues samples	–	Enhancer	[79]
	Qin et al./2018	MMP-9	Suppressed proliferation, migration and invasion, and promoted apoptosis	**↓**	Tissues samples/cell lines	lncRNA-01296	Suppressor	[114]
Bile duct	Xu et al./2018	ALDOA	Inhibited proliferation and invasion	↓	Cell culture/tissue samples	–	Suppressor	[29]
	Kong et al./2019	CLIC1	Inhibited Bile duct carcinoma progression	↓	Cell line	LncRNA-UCA1	Suppressor	[30]
	Zhu et al./2016	–	Inhibited cell proliferation	↓	Cell culture/tissue samples	–	Suppressor	[31]
	Loosen et al./2019	–	Promising tool for the diagnosis of even early stage CCA	↑	Serum samples	–	Enhancer	[73]
Colorectal	Wang et al./2020	PKM2	Reversion of oxaliplatin resistance in CRC	↓	Cell culture/tissue samples	hsa_circ_0005963	Suppressor	[83]
	Yin et al./2020	CDC25A	Inhibited CRC progression	↓	Cell culture/tissue samples	circ_0007142	Suppressor	[32]
	Sun et al./2020	–	As a potential diagnostic and prognostic biomarker	↑	Serum samples/Cell culture	–	Enhancer	[33]
	Maierthaler et al./2017	–	As a potential prognostic biomarker	↑	Plasma samples	–	Enhancer	[80]
Colon	Li et al./2019	ALDOA	Promotes the proliferation and invasion	↑	Cell line	–	Enhancer	[34]
Esophageal	Wang et al./2021	KIF22	Blocked cell proliferation, migration and invasion	**↓**	Cell culture/tissue samples	–	Suppressor	[35]
	Zhang et al./2016	PKM2	Functioned on growth inhibition	**↓**	Cell line	–	Suppressor	[36]
Gallbladder	Lu et al./2016	PKM2	Inhibits cancer cell malignancy	**↓**	Cell line/Tissue samples	–	Suppressor	[37]
Nasopharyngeal	Liu et al./2019	SATB1	Suppresses cell proliferation, migration and invasion	**↓**	Cell Line	–	Suppressor	[38]
Breast	Fong et al./2015	PKM	Reprograms glucose metabolism in premetastatic niche to promote metastasis	**↑**	Cell culture/tissue samples	–	Enhancer	[39]
	Wang et al./2020	CHMP3	promotes aggression and EMT in TNBC	**↑**	cell line	–	Enhancer	[40]
	Wang et al./2012	IGF1R	Plays an important role in inhibiting the tumorigenesis	↓	Cell culture/tissue samples	–	Suppressor	[41]
	Zhang et al./2019	Bcl-2/CDKs	Enhance the chemosensitivity of breast cancer	**↑**	Cell line	–	Enhancer	[88]
	Perez‐Añorve et al./2019	ZNF611/ZNF304	Controls the response to radiotherapy	**↑**	Cell line	–	Enhancer	[86]
	Qin et al./2021	STK39	Inhibited breast cancer progression	↓	Cell culture/tissue samples	LncRNA-CDKN2B-AS1	Suppressor	[42]
	Uen et al./2018	syndecan-1	Increased breast cancer cell mobility	**↑**	Cell line	–	Enhancer	[115]
	Zhang et al./2018		Inhibited breast cancer progression	↓	Cell line	LncRNA-RPPH1	Suppressor	[116]
	Ergün et al./2015	ADAM10	Potential regulator of trastuzumab resistance	↓	Tissue samples	–	Suppressor	[117]
	Saleh et al./2019	–	ASSOCIATED with decreased overall survival and progression-free survival	**↑**	Whole blood samples	–	Enhancer	[118]
Ovarian	Duan et al./2018	P4HA1	Inhibited migration, invasion, and EMT	**↓**	Cell culture/tissue samples	–	Suppressor	[43]
	Huang et al./2022	SLC1A5	Suppressed cell proliferation, migration, invasion and accelerated cell apoptosis	**↓**	Cell culture/tissue samples	Circ_0072995	Suppressor	[44]
Bladder	Wang et al./2016	VEGFC	Regulated cell proliferation	**↓**	Cell culture/tissue samples	–	Suppressor	[45]
	Guo et al./2018	CREB1	Regulated cell proliferation and invasion	**↓**	Cell culture/tissue samples	–	Suppressor	[46]
Cervical	Ding et al./2019	CDC25A	Modulates the radiosensitivity of cervical cancer cells	**↓**	Cell culture/tissue samples	–	Suppressor	[87]
	Yang et al./2022	RAD21	Block malignant growth and promoted apoptosis	**↓**	Cell culture/tissue samples	–	Suppressor	[47]
Pancreatic	Hu et al./2021	–	Suppressed cell proliferation and invasion	**↓**	Cell line/tissue samples	LncRNA PART1 p	Suppressor	[48]
	Yin et al./2020	XIAP	Restraining tumorigenic ability	**↓**	Cell line	LncRNA SBF2-AS1	Suppressor	[49]
	Dai et al./2020	CCNG1	Inhibit cell proliferation, migration, invasion, and EMT	**↓**	Cell line/tissue samples	–	Suppressor	[50]
	Mazza et al./2020	–	Associated with worse prognosis and clinical outcome	↑	Plasma samples	–	Enhancer	[74]
	Cui et al./2018	ALDOA	Blocked proliferation and invasion	**↓**	Cell line	lncRNA DIO3OS	Suppressor	[119]
Prostate	Liu et al./2019	ROCK2	Inhibit cell proliferation	**↓**	Cell line/serum samples	–	Suppressor	[51]
	Zhu et al./2020	PKM2	Reverse the resistance of LNCaP/Docetaxel cells to docetaxel	**↓**	Cell line	–	Suppressor	[90]
Thyroid	Hu et al./2021	DUSP4	inhibited the proliferation, invasion, and migration	**↓**	Cell line/tissue samples	–	Suppressor	[52]
Renal	Wang et al./2019	PKM2	Promotes cancer cell viability, proliferation, migration, glycolysis and autophagy	**↑**	Cell line	–	Enhancer	[53]
	Lian et al./2013	–	Promotes proliferation, invasion and migration	**↑**	Cell line	–	Enhancer	[54]
	Wang et al./2017	Sprouty2	Act as a tumor promoter	**↑**	Cell line/tissue samples	–	Enhancer	[55]
	Fan et al./2018	Dicer	Promotes metastatic behavior	**↑**	Cell line/tissue samples	–	Enhancer	[56]
	Heinemann et al./2018	–	High serum miR-122-levels indicate advanced stage/grade	**↑**	Serum samples	–	Enhancer	[57]
	Cochetti et al./2022		Aid in the early diagnosis	**↑**	Urinary samples		Enhancer	[120]
Lung	Li et al./2021	–	Mediated lung cancer progression	↑	Cell line	–	Enhancer	[58]
	Gao et al./2020	FSTL3	Reversed the proliferation and metastasis	**↓**	Cell line/tissue samples	lncRNA DSCAM-AS1	Suppressor	[59]
	Ma et al./2015	–	Induces radiosensitization	**↓**	Cell line	–	Suppressor	[60]
	Chandimali et al./2019	Prx II	inhibited cancer stem cell proliferation\	**↓**	Cell line	–	Suppressor	[61]
	Zhao et al./2015	CCNG1/MEF2D	Induced cell cycle arrest	**↓**	Cell line/tissue samples	–	Suppressor	[121]
Osteosarcoma	Ma et al./2021	–	Inhibited the proliferation, invasion, and migration	**↓**	Cell line/tissue samples	LINC01410	Suppressor	[62]
	Li et al./2020	TP53	Inhibit the proliferation and promote the apoptosis of osteosarcoma cells	**↓**	Cell line/tissue samples	–	Suppressor	[63]
	Yuan et al./2020	ADAM10	Induced inhibition of cell proliferation, migration, and invasion	**↓**	Cell line	–	Suppressor	[64]
Glioma	Sun et al./2018	–	Repressed the Proliferation, Migration, and Invasion	**↓**	Cell line/tissue samples	lncRNA UCA1	Suppressor	[67]
	Wang et al./2014	WNT1	Repressed the Proliferation	**↓**	Cell line/tissue samples	–	Suppressor	[68]
	Tang et al./2017	–	Function as a diagnostic and prognostic biomarker	**↓**	Plasma samples	–	Suppressor	[75]
Hematological	Yang et al./2017	–	Serve as a prognostic factor in AML	**↓**	Cell culture/tissue samples	–	Suppressor	[69]
	Beg et al./2021	–	Involved in imatinib response in CML patients	**↓**	tissue samples	–	Suppressor	[70]
Melanoma	Li et al./2018	NOP14	Inhibits the proliferation	↑	Cell culture/tissue samples	–	Enhancer	[71]
Oral squamous cell carcinoma	Tian et al./2021	RhoA	Blocked cell invasion and migration	**↓**	Cell line/tissue samples	LINC00974	Suppressor	[72]

## Data Availability

The datasets used and/or analyzed during the current study are available from the corresponding author on reasonable request.

## Abbreviations

MRNAs: Messenger RNAs
MiRNAs: MicroRNAs
Nt: Nucleotide
pri-miRNAs: Primary miRNAs
XPO5: Exportin 5
AGO: Argonaute
RISC: RNA-induced silencing complex
3ʹ-UTR: 3ʹ-Untranslated region
HNF-4: Hepatocyte nuclear factor 4
HNF4A: Hepatocyte nuclear factor 4A
NIK: NF-кB-inducing kinase
NAFLD: Non-alcoholic fatty liver disease
AMPK: AMP-activated protein kinase
HCC: Hepatocellular carcinoma
G6PD: Glucose-6-phosphate dehydrogenase
LMNB2: Lamin B2
APOBEC2: Apolipoprotein B mRNA editing enzyme catalytic polypeptide 2
DNMTs: DNA Methyltransferases
LC: Liver cancer
GC: Gastric cancer
OS: Overall survival
DUSP4: Dual specificity phosphatase 4
CREB1: CAMP response element binding protein 1
DM: Distant metastasis
AFP: Fetoprotein
AFPGC: AFP-producing gastric cancer
FOXO3: Forkhead box O3
CCA: Cholangiocarcinoma
BDC: Bile duct cancer
ALDOA: Aldolase A
MAPK: Mitogen-activated protein kinase
ERK: Extracellular signal-regulated kinase
CRC: Colorectal cancer
CDC25A: Cell division cycle 25A
LM: Liver-metastasized
KIF22: Kinesin family member 22
ESCC: Esophageal squamous cell carcinoma
PKM2: Pyruvate kinase isozymes M2
GBC: Gallbladder cancer
EMT: Epithelial-mesenchymal transition
NPC: Nasopharyngeal carcinoma
SATB1: Sequence-binding protein 1
BC: Breast cancer
TNBC: Triple-negative breast cancer
PI3K: Phosphoinositide 3-kinase
mTOR: Mammalian target of rapamycin
p70S6K: P70S6 kinase
IGF1R: Insulin like growth factor1 receptor
STK39: Serine/threonine kinase 39
OC: Ovarian cancer
P4HA1: Prolyl 4-hydroxylase subunit alpha 1
VEGFC: Vascular endothelial growth factor C
CREB1: CAMP responsive element binding protein 1
CC: Cervical cancer
PanC: Pancreatic cancer
PCa: Prostate cancer
ROCK2: Rho associated coiled-coil containing protein kinase 2
PDAC: Pancreatic ductal adenocarcinoma
PTC: Papillary thyroid cancer
RC: Renal carcinoma
ccRCC: Clear-cell renal cell carcinoma
EVs: Extracellular vesicles
NSCLC: Non-small cell lung cancer
FSTL3: Follistatin like 3
CSCs: Cancer stem cells
OS: Osteosarcoma
UCA1: Urothelial carcinoma-associated 1
ADAM10: ADAM metallopeptidase domain 10
AML: Acute myeloid leukemia
CML: Chronic myelogenous leukemia
OSCC: Oral squamous cell carcinomas
XIAP: X-linked inhibitor of apoptosis protein
DOX: Doxorubicin
LDHA: Lactate dehydrogenase A
PDK4: Pyruvate dehydrogenase kinase 4
GSCs: Glioblastoma stem cells
MSCs: Mesenchymal stem cells
AMSC: Adipose tissue derived MSC
GCs: Granulosa cells
PAMAM: Polyamidoamine
Gal-LCP: Galactose-targeted lipid calcium phosphate
GGMPN: Graphene nanocomposites
P-gp: P-glycoprotein
ciRS-122: hsa_circ_0005963 was a sponge for miR-122 and named ciRS-122
